# *Ascaris* co-infection does not alter malaria-induced anaemia in a cohort of Nigerian preschool children

**DOI:** 10.1186/1475-2875-12-1

**Published:** 2013-01-02

**Authors:** Francisca A Abanyie, Courtney McCracken, Patrick Kirwan, Síle F Molloy, Samuel O Asaolu, Celia V Holland, Julie Gutman, Tracey J Lamb

**Affiliations:** 1 Emory University School of Medicine, Atlanta, GA, 30322, USA; 2 Biostatistics Core, Emory University and Children’s Pediatric Research Centre, Atlanta, GA, 30322, USA; 3 Department of Zoology, School of Natural Sciences, Trinity College Dublin, Dublin 2, Ireland; 4 Department of Zoology, Obafemi Awolowo University, Ile-Ife, Nigeria

## Abstract

**Background:**

Co-infection with malaria and intestinal parasites such as *Ascaris lumbricoides* is common. Malaria parasites induce a pro-inflammatory immune response that contributes to the pathogenic sequelae, such as malarial anaemia, that occur in malaria infection. *Ascaris* is known to create an anti-inflammatory immune environment which could, in theory, counteract the anti-malarial inflammatory immune response, minimizing the severity of malarial anaemia. This study examined whether *Ascaris* co-infection can minimize the severity of malarial anaemia.

**Methods:**

Data from a randomized controlled trial on the effect of antihelminthic treatment in Nigerian preschool-aged (6–59 months) children conducted in 2006–2007 were analysed to examine the effect of malaria and *Ascaris* co-infection on anaemia severity. Children were enrolled and tested for malaria, helminths and anaemia at baseline, four, and eight months. Six hundred and ninety subjects were analysed in this study. Generalized linear mixed models were used to assess the relationship between infection status and *Ascaris* and *Plasmodium* parasite intensity on severity of anaemia, defined as a haemoglobin less than 11 g/dL.

**Results:**

Malaria prevalence ranged from 35-78% over the course of this study. Of the malaria-infected children, 55% were co-infected with *Ascaris* at baseline, 60% were co-infected four months later and 48% were co-infected eight months later, underlining the persistent prevalence of malaria-nematode co-infections in this population. Over the course of the study the percentage of anaemic subjects in the population ranged between 84% at baseline and 77% at the eight-month time point. The odds of being anaemic were four to five times higher in children infected with malaria compared to those without malaria. *Ascaris* infection alone did not increase the odds of being anaemic, indicating that malaria was the main cause of anaemia in this population. There was no significant difference in the severity of anaemia between children singly infected with malaria and co-infected with malaria and *Ascaris*.

**Conclusion:**

In this cohort of Nigerian preschool children, malaria infection was the major contributor to anaemia status. *Ascaris* co-infection neither exacerbated nor ameliorated the severity of malarial anaemia.

## Background

Malaria continues to plague millions of individuals annually, with an estimated 216 million cases reported each year and an estimated 655,000 deaths in 2010, 86% of which occurred in children under five years of age [1]. Anaemia is one of the main complications of malaria infection, and most severely affects children between one and three years of age in areas where there is high transmission of *Plasmodium falciparum*[2]. The effects of long-standing or severe anaemia can be devastating and include impairment of physical and cognitive development, especially in association with iron-deficiency; additionally, severe anaemia has been associated with an increased risk of death [3]. The World Health Organization (WHO) estimates that approximately 67.6% of preschool-age children in Africa are anaemic [3], which places this region at the highest severity of public health significance.

The cause of severe malarial anaemia (SMA) is multifactorial. Evidence from human malaria infections, as well as animal models of malaria, suggest that destruction of red blood cells (RBCs) by malaria parasite proliferation and clearance of malaria-infected red blood cells (iRBCs) by the immune system are only contributory factors to the severity of malarial anaemia [4,5]. It is now accepted that the effects of malaria infection on the immune system can directly cause anaemia in malaria-infected individuals by altering levels of circulating uninfected RBCs [6-8].

Children suffering from malaria infection are often also co-infected with intestinal helminth infection. Intestinal helminths remain the most common infection worldwide with over 230 million preschool-aged children affected. *Ascaris lumbricoides* accounts for the majority of these infections, with an estimated one billion individuals infected [9]. *Ascaris* infection induces anti-inflammatory Th2 responses [10-12] and is also associated with an immunoregulatory immune response defined by elevated levels of interleukin (IL)-10 and transforming growth factor-β (TGF-β) [13,14].

The immune response to *Ascaris* can down-regulate pro-inflammatory cytokines and suppress unrelated immune responses such as those to vaccination [14] or simultaneous infection [14,16]. Indeed such immune interaction is thought to be responsible for the protective effect against cerebral malaria, malaria-related jaundice and renal failure that has been reported [17,18]. Since malarial anaemia has a strong pro-inflammatory component, and *Ascaris* infection induces immunoregulation that can interact with unrelated immune responses, one might expect that a concurrent *Ascaris* infection could decrease the severity of malarial anaemia.

This study was undertaken to explore the effect of *Ascaris* infection on malarial anaemia in a cohort of Nigerian preschool-age children.

## Methods

### Study population and design

The description of the study population, design, and methods of the original study are detailed in prior publications [19,20]. Briefly, a double-blind, randomized, placebo-controlled trial was conducted in four semi-urban villages situated near Ile-Ife, in Osun State, Nigeria. The goal of the study was to investigate the impact of repeated antihelminthic therapy with albendazole on *Plasmodium* infection in children between 12 and 60 months of age. Children were randomized to receive either placebo or albendazole every four months during the study period. Stool specimens were examined for the presence of helminth infections. Eggs per gram (epg) of faeces was used to estimate parasite intensity. Finger-prick blood specimens were taken for analysis of *Plasmodium* infection via malaria rapid diagnostic testing (RDT) (Parascreen, Zephyr Biomedicals, Verna Industrial Estate, Verna Goa, India) and microscopic examination for malaria parasites. Haemoglobin levels were determined using a haemoglobinometer (Accuscience, Ireland). Children suffering from a malaria attack were treated with artemether-lumefantrine.The study was approved by the Ethics and Research Committee, Obafemi Awolowo University Teaching Hospital’s Complex, Ile-Ife, Nigeria. Informed consent was obtained from the mother of each child included in this study.

This study utilized the available data from the baseline, four-month, and eight-month time points. At each time point, only those patients with a result for haemoglobin, malaria testing, and stool microscopy for helminths were included. Mild anaemia was defined as haemoglobin (Hb) level between 10.0 and 10.9 g/dl, moderate anaem ia was defined as 7.0 g/dL≤ Hb <10 g/dL, and severe anaemia was defined as Hb <7.0 g/dL [21].

### Statistical analysis

Statistical analysis was performed using SAS 9.2 (Cary, NC, USA). Statistical significance was assessed at the 0.05 level unless otherwise noted. Infection groups were defined by the presence or absence of *Ascaris* infection and/or malaria infection at each time point, resulting in the following four groups: (1) no infection; (2) infection with malaria only; (3) infection with *Ascaris* only; and, (4) infection with both *Ascaris* and malaria (co-infection). Haemoglobin values were analysed as a continuous variable and then categorized into multinomial responses for mild, moderate or severe anaemia.

The categorization of haemoglobin allowed for calculations of odds ratios between infection groups. Univariate analysis was performed to calculate descriptive statistics of the predictors and assess normality of the outcome variables. Due to the highly skewed distribution of parasite intensity in children, the ranks of the data were used in place of the actual values of parasite intensity. The ranks were determined by ordering the data from least to greatest and replacing each observation by its relative position in the order. Assuming no ties, the smallest parasite intensity would receive a rank of 1 while the largest intensity would receive a value of *n* (the number of observations). The demographics from each infection group were compared at baseline to describe the overall sample of children. Comparisons between the four groups were made using chi-squared tests, analysis of variances models, or Kruskal-Wallis tests. The Tukey-Kramer multiple comparison procedure was used to determine the significance of pair-wise comparisons.

Parasite intensity and haemoglobin levels were analysed using repeated measures linear mixed models using the MIXED procedure in SAS to account for unbalanced data and multiple observations per child. An infection group-by-time variable was initially included in the haemoglobin model but was removed after it failed to retain significance. Similarly, an anaemia severity group-by-time variable was included in the parasite intensity model but was not retained. The original study treated children with albenzadole or placebo according to the study design; however this factor was removed from the model because it failed to show an association with anaemia severity and did not significantly change the estimates in the model. Means and standard deviations were used to describe haemoglobin levels within each group. Medians and ranges were used to describe the parasite intensities within each anaemia severity group.

The effect of infection group on anaemia severity was analysed with generalized linear mixed models using a cumulative logit function with the assumption that the data are from a multinomial distribution. The link function allows for the ordinality of the outcome variable anaemia severity. Thus the parameter estimates represent the log odds of being anaemic *vs* normal and the estimates have been transformed to odds ratios for ease of interpretation. A random effect for each individual was added to the model to account for the repeated measures study design. The effect of sex, age, and SES were also included in the model. Since hookworm infection is known to cause anaemia [22], the model adjusted for the effect of hookworm in our initial model; however, this was dropped because this factor conferred little to no change in the odds ratio estimates.

## Results

### Co-infection with malaria and *Ascaris* was common in this cohort of individuals

Of a total 690 children analysed at baseline, 245 (35.5%) subjects were singly infected with malaria parasites; 72 (10.4%) singly infected with *Ascaris*; 296 (42.9%) were co-infected with malaria and *Ascaris* and 77 (11.2%) subjects were uninfected with either malaria or *Ascaris* (Table 1). When all three time points were considered, the total number of children infected with malaria on at least one occasion was 541 (78.4%); the total number of children infected with *Ascaris* on at least one occasion was 368 (53.3%); and 613 (88.8%) were infected with at least one parasite (either malaria or *Ascaris*). The proportion of patients with malaria was not significantly different between children infected with the *Ascaris* and children with no detectable *Ascaris* infection (p=0.166). Infection with other intestinal helminths was rare. The prevalence of hookworm and schistosomiasis was 5.2% and 0.6% respectively at baseline and 4.1% and 1.0% when all time points were considered. There were 98 instances of hookworm infection: 36 at baseline, 48 at four months, and 14 at eight months.

**Table 1:** Baseline characteristics-comparing baseline infection groups

**Characteristic**	**Level**	**Infection group**				**p-value**
		**No infection**	**Ascaris only**	**Malaria only**	**Co-infection**	
		**(N = 77)**	**(N = 72)**	**(N = 245)**	**(N = 296)**	
Age (months) Mean ± SD		34.0 ± 13.9	34.4 ± 11.6	33.4 ± 13.1	35.5 ± 12.9	0.332
Age (years)	*1*	26 (34.7%)	15 (21.7%)	64 (28.1%)	63 (22.5%)	0.078
	*2*	15 (20.0%)	22 (31.9%)	71 (31.1%)	82 (29.3%)	
	*3*	16 (21.3%)	24 (34.8%)	48 (21.1%)	74 (26.4%)	
	*4*	18 (24.0%)	8 (11.6%)	39 (17.1%)	57 (20.4%)	
	*5*	0 (0.0%)	0 (0.0%)	6 (2.6%)	4 (1.4%)	
Sex	*Male*	38 (50.0%)	33 (45.8%)	123 (51.3%)	156 (52.7%)	0.767
	*Female*	38 (50.0%)	39 (54.2%)	117 (48.7%)	140 (47.3%)	
SES-Indexª Mean ± SD		13.1 ± 2.5	12.5 ± 2.1	12.1 ± 2.6	11.9 ± 2.4	0.003
Weight-for-height z-score (WHZ) Mean ± SD		−0.66 ± 0.91	−0.66 ± 0.94	−0.69 ± 0.88	−0.59 ± 0.87	0.636
Haemoglobin (g/dL) Mean ± SD		10.1 ± 1.4	10.1 ± 1.5	9.0 ± 1.5	9.3 ± 1.6	< 0.001
Anaemia	Normal Hb	22 (28.6%)	22 (30.6%)	20 (8.2%)	45 (15.2%)	< 0.001
	Mild	24 (31.2%)	19 (26.4%)	48 (19.6%)	57 (19.3%)	
	Moderate	30 (39.0%)	30 (47.7%)	159 (64.9%)	173 (58.5%)	
	Severe	1 (1.3%)	1 (1.3%)	18 (7.4%)	21 (7.1%)	

ª Socio-economic status.

### Anaemia was associated with malaria infection but not *Ascaris* infection

**Figure 1:**
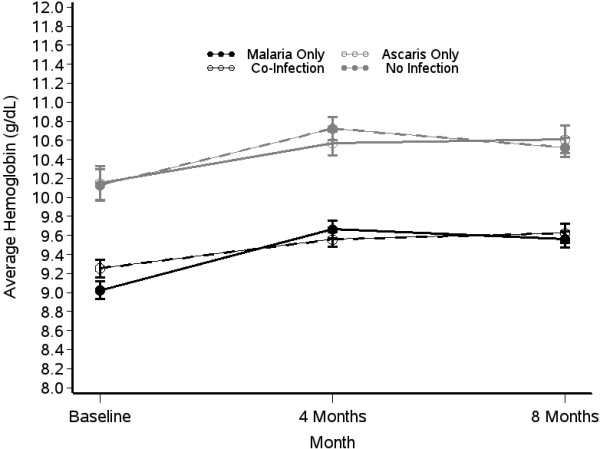
**Change in the average haemoglobin levels of children who were infected with malaria (black line, circular symbols), *Ascaris *(grey lines open symbols) or co-infected with both parasites (black line, star symbols) over time.** Children with no detectable infection of either parasite (grey line, closed symbols) are shown for comparison. Symbols show the mean haemoglobin levels and the error bars represent the standard error of the mean.

The overall prevalence of anaemia (Hb <11 g/dL) at baseline was 84%; 63% of those with anaemia were considered to have moderate or severe disease. For each infection group there was an increase in the average haemoglobin from baseline to four months and small changes from four months to eight months (Figure 1). The change in haemoglobin over time was the same in each group (p = 0.46).

The effect of infection group on severity of anaemia was assessed at all three time points. Overall, there were significant differences in haemoglobin values of children with malaria infection and children without malaria infection. At baseline, children with malaria infection had lower haemoglobin values compared to children with *Ascaris*-only infection or no infection (p < 0.001 for both comparisons) (Figure 1, Table 1). Overall, the odds of being anaemic (mild, moderate, or severe) were highest in malaria infection groups compared to non-malaria infection groups. Anaemia was more common in children infected only with malaria than in children infected only with *Ascaris* or in uninfected children [odds ratio (OR), 3.67; 95% CI, 2.23–6.07 and OR, 3.77; 95% CI, 3.77–6.12, respectively] (Table 2).

**Table 2:** Odds ratios: modelling the probability of having anaemia (haemoglobin <11 g/dL) at baseline

**Crude odds ratio (95% confidence interval) of anaemia (haemoglobin <11 g/dL) at baseline**				
**Infection group**	Malaria	*Ascaris* only	Co-infection	No infection
Malaria only		3.67 (2.23 – 6.07)*	1.37 (0.97 – 1.91)	3.77 (2.32 – 6.12)*
*Ascaris* only			0.37 (0.23 – 0.61)*	1.03 (0.57 – 1.85)
Co-infection				2.76 (1.73 – 4.41)*
No infection				
* p-value <0.001				

### Malaria parasite intensity was positively correlated with the severity of anaemia

Anaemia prevalence and degree of anaemia were significantly correlated with increasing malaria parasite intensity (Table 3). The highest median *Plasmodium* intensities at baseline were seen in moderate and severe anaemia groups (Table 3). In both cases, anaemic children had significantly higher *Plasmodium* parasite intensities than children without anaemia (severe anaemia *vs* no anaemia p = 0.002; moderate anaemia *vs* no anaemia p = 0.017). Severely anaemic children also had a trend towards higher levels of *Plasmodium* parasites than children with mild anaemia, but the p-value was no longer significant after adjusting for multiple comparisons (p = 0.09). Similar results between anaemia groups were found at four months and eight months.

**Table 3:** Comparison of parasite intensity across infection groups (at baseline)

**Parasite intensity**	**Degree of anaemia**				**P-value**
Malaria	Normal Hb	Mild	Moderate	Severe	0.009
	(N = 56)	(N = 72)	(N = 179)	(N =16)	
	0	0	200	2,020	
	(0 – 14,960)	(0 – 21,240)	(0 – 55,280)	(0 – 16,960)	
Helminths	Normal Hb	Mild	Moderate	Severe	0.339
	(N = 67)	(N = 82)	(N = 212)	(N = 23)	
	450	236	389	742	
	(1 – 27,560)	(1 – 15,960)	(1 – 23,476)	(3 – 11,239)	
*Ascaris*	Normal Hb	Mild	Moderate	Severe	0.445
	(N = 67)	(N = 76)	(N = 203)	(N = 22)	
	450	277.5	434	765	
	(1– 27,560)	(1 – 15,960)	(1 – 23,421)	(3 – 11,239)	

The median intensity of malaria parasites, helminth parasites (*Ascaris*, *Schistosoma Trichuris* and hookworm) for each anaemia group at baseline is shown.

### Helminth parasite intensity was not correlated with severity of anaemia

Helminth parasite intensity (including hookworm, *Schistosoma* and *Trichuris* in addition to *Ascaris*) was examined in each anaemia group over time. As expected, parasite intensities changed over time (results not shown) but were not significant when comparing anaemia groups (Table 3). Furthermore there was no significant correlation between parasite density and anaemia prevalence or degree of anaemia when all worms were included (p = 0.13) or when infection with *Ascaris-*only was considered (p = 0.20). Hookworm infection was not significantly associated with an increased odds of anaemia at baseline (p = 0.59) or at any of the three time points analysed (p = 0.66).

### The severity of malarial anaemia is not altered by the presence of *Ascaris* co-infection

Children with malaria-*Ascaris* co-infection did not have significantly different haemoglobin levels (p = 0.306) or malaria parasite densities (p=0.965) than children with malaria-only infection but did have significantly lower haemoglobin levels than uninfected or *Ascaris*-only infected children (p<0.001) (Table 4). Furthermore children with *Ascaris*-only infection had haemoglobin levels that did not significantly differ from children with no infection (p = 0.999). Similar results were seen at four months and eight months. The odds of having anaemia were greater in the co-infection group when compared to the uninfected and *Ascaris*-only group, but were the same when compared to the malaria only group (Table 2). This study assessed whether other factors known to be involved in mediating susceptibility to malaria infection and pathology such as age [23] or social economic status [24,25] may have masked the impact of helminth infection on anaemia. Both younger age and lower socio-economic status were associated with increased odds of anaemia. However, even after adjusting for these potential confounders, *Ascaris* remained an insignificant variable of the severity of malarial anaemia (p = 0.60) (Table 4).

**Table 4:** Generalized linear mixed effects model for the severity of anaemia in children

**Demographic**		**Estimate**	**SE**	**Odds ratio ª**	**P-value**
Infection group ‘b’	Malaria only	1.72	0.17	5.63	< 0.001
	*Ascaris only*	−0.11	0.211	0.90	0.601
	Co-infection	1.43	0.17	4.17	< 0.001
Age	--	−0.041	0.005	0.96	< 0.001
Time	--	−0.174	0.067	0.84	0.009
SES	--	−0.078	0.027	0.92	0.003
Males	--	0.201	0.132	1.22	0.130

‘b’ Reference group = no infection.

ª The odds represent *Pr*(anemic)/(1-*Pr*(anaemic)).

## Discussion

Anaemia was highly prevalent in this cohort of preschool Nigerian children, with a rate of 84%. This is significantly higher than prior studies [26,28], and exceeds WHO’s estimate for anaemia among preschool-age children in Africa [21]. Understanding the causal factors is crucial to developing an effective intervention. As has been shown previously, malaria infection was significantly associated with anaemia. Co-infection with *Ascaris* and malaria was common in this cohort of children and this study investigated whether the severity of malarial anaemia was altered in co-infected children.

The initial hypothesis was that *Ascaris* infection would be protective with respect to the severity of malarial anaemia. The rationale for this hypothesis was that the induction of an anti-*Ascaris* immunosuppressive response would dampen the anti-malarial inflammatory immune responses, thus reducing the contribution of the inflammatory immune response on malarial anaemia [29]. However, it was found that concurrent infection with *Ascaris* did not have any effect on the severity of malarial anaemia, in agreement with a previous study carried out in Cameroon [30], and in partial agreement with a study looking at hookworm and malaria interaction in Zanzibar [31]. In this study, there were slightly more anaemic children in the malaria-only infected group compared to the co-infected group (92% vs. 85%, respectively) but the odds ratio was not statistically significant and fell short of clinical significance.

Circulating levels of tumour necrosis factor (TNF) [32], macrophage migration inhibitory factor (MIF) [33] and interleukin-12 (IL-12) [34] correlate with the severity of malarial anaemia, and have been demonstrated to play a role in causing malarial anaemia in animal models [33,35]. These pro-inflammatory cytokines lower the production of erythropoietin, the renal hormone responsible for RBC formation, thereby leading to anaemia [36-38]. Animal models of malaria suggest that erythroid suppression is further exacerbated by a reduced response of progenitor cells to erythropoietin [39,40].

The immunoregulatory cytokine IL-10 is a powerful antagonist to the contributory effects of TNF on malarial anaemia. An elevated ratio of IL-10 to TNF in the serum is associated with a decreased severity of anaemia in Kenyan children [41]. Immunoregulatory immune responses in malaria infection [42,43] can down-regulate the production and action of pro-inflammatory cytokines [44-47]. Although in co-infected children there is potential for helminth-induced immunoregulatory cytokines to reduce the magnitude of the pro-inflammatory immune response induced by malaria infection, there may not be any visible effect if most pre-school children are already asymptomatic because of a prevailing malaria-induced immunoregulatory response. The majority of the children included in this study were otherwise asymptomatic from malaria infection [20]. This phenomenon has previously been noted in older Nigerian school-age children by Ojurongbe *et al.*[48] indicating that the immunoregulatory properties of malaria infection can develop quickly in preschool-age children.

The causes of anaemia in children living in resource poor settings are numerous. In this analysis, age and socio-economic status were significantly correlated with anaemia. Periods of rapid growth including early childhood (from the prenatal period to eight years of age), as in this cohort, and adolescence are notable for increased iron utilization which can lead to iron-deficiency and, in many cases, associated anaemia [49].

Other factors that were not analysed may also contribute to anaemia, masking the effect of co-infection on the severity of malarial anaemia. Nutritional factors such as vitamin and iron deficiencies play a significant role in the development of anaemia in resource poor regions of the world, like Nigeria. Additionally, sickle cell disease [50,51] and other haemoglobinopathies [52] as well as HIV status [53,54] may be contributing factors to the severity of anaemia.

## Conclusion

In this cohort, anaemia was highly prevalent and correlated with malaria infection status. *Ascaris* co-infection had no effect on malarial anaemia status or severity. While de-worming is crucial, this study suggests that ensuring that preschool children have adequate access to diagnosis and treatment of malaria is paramount in protecting them from the devastating effects of severe anaemia. This study highlights the continued need for research and resource allocation to efforts targeted towards the elimination of malaria worldwide. Despite these findings, the importance of de-worming should not be overlooked as the devastating effects on growth and nutritional status that helminths produce cannot be understated.

## Abbreviations

CI: Confidence interval; Hb: Haemoglobin; HIV: Human immunodeficiency virus; IL: Interleukin; iRBC: Infected red blood cell; OR: Odds ratio; RBC: Red blood cell; RDT: Rapid diagnostic test; SMA: Severe malarial anaemia; TGF-β: Transforming growth factor-β; Th2: T-helper 2 cells; TNF: Tumour necrosis factor.

## Competing interests

The authors declare that they have no competing interests.

## Authors’ contributions

FA participated in the interpretation of the data and drafted the manuscript. CM performed statistical analysis of the data and drafted the manuscript. PK, SM, and SA coordinated the study. CH designed and coordinated the study. JG conceived the design of the study, participated in the interpretation of the data and drafted the manuscript. TL conceived design of the study, participated in the interpretation of the data and drafted the manuscript. All authors read and approved the final manuscript.
